# Bio-Based Poly(3-hydroxybutyrate) and Polyurethane Blends: Preparation, Properties Evaluation and Structure Analysis

**DOI:** 10.3390/ma18091914

**Published:** 2025-04-23

**Authors:** Beata Krzykowska, Anna Fajdek-Bieda, Aneta Jakubus, Joanna Kostrzewa, Anita Białkowska, Maciej Kisiel, Štěpánka Dvořáčková, Wiesław Frącz, Iwona Zarzyka

**Affiliations:** 1 Department of Organic Chemistry, Faculty of Chemistry, Rzeszów University of Technology, Powstańców Warszawy 6, 35-959 Rzeszów, Poland; 2 Department of Medical Analytics, Faculty of Health Sciences, The Jacob of Paradies University in Gorzów Wielkopolski, Chopina 52, 66-400 Gorzów Wielkopolski, Poland; abieda@ajp.edu.pl; 3 Faculty of Technology, Jacob of Paradies University, 66-400 Gorzów Wielkopolski, Poland; jakubusaneta@wp.pl (A.J.); kostrzewa_joanna@wp.pl (J.K.); 4 Faculty of Mechanic, Radom University, Stasieckiego 54, 26-600 Radom, Poland; a.bialkowska@uthrad.pl; 5 Department of Industrial and Materials Chemistry, Faculty of Chemistry, Rzeszów University of Technology, Powstańców Warszawy 6, 35-959 Rzeszów, Poland; m.kisiel@prz.edu.pl; 6 Department of Machining and Assembly, Technical University of Liberec, Studentská 1402/2, 460 01 Liberec, Czech Republic; stepanka.dvorackova@tul.cz; 7 Department of Material Forming and Processing, Rzeszów University of Technology, Powstańców Warszawy 8, 35-959 Rzeszów, Poland; wf@prz.edu.pl

**Keywords:** polymer blends, polyhydroxyalkanoates, polyurethanes, structure-properties relationship, thermal stability, mechanical properties, structure

## Abstract

The present work deals with polymer blends produced from poly(3-hydroxybutyrate), P3HB and polyurethane. Linear polyurethane (PU) was here synthesized by reacting polypropylene glycol with 4,4′-diphenylmethane diisocyanate, and was used in amounts of 5, 10 and 15 wt. %. The polymers were melt-mixed using a twin-screw extruder after prior premixing. The obtained blends were tested by differential scanning calorimetry analysis (DSC), Fourier transformation infrared spectroscopy (FTIR), and scanning electron microscopy (SEM) with energy dispersive X-ray analysis (EDX). Their thermal and mechanical properties, including impact resistance, hardness, tensile and flexural properties, were also determined, and the surface topography and roughness were analyzed. FTIR analysis of the prepared blends confirmed the interactions of PU with the P3HB matrix via hydrogen bonding. Analysis of the surface topography of the samples showed that the higher the PU content, the greater the regularity and homogeneity of the surface structure. The roughness of the P3HB blend containing 5 wt. % PU was the greatest. SEM images of the fracture surfaces of the blend samples explain the mechanism of the improvement of their mechanical properties. The obtained polymer blends were characterized by significantly lower hardness, and better impact strength and relative elongation at break compared to native P3HB. The DSC results confirm a decrease in the glass transition, melting and crystallization temperatures with increasing amounts of PU in the blends. The lower melting temperature and the higher degradation temperature of the resulted blends than native P3HB make the processing conditions easier, and prevent the degradation of the material. The best mechanical and thermal properties were shown by blends containing 10 wt. % of PU.

## 1. Introduction

One of the biggest problems of the current era is recycling and reducing the amount of plastic materials; for this reason, many scientists are developing completely biodegradable, non-toxic and nature-friendly polymeric materials. The biopolymers being produced as biodegradable and biosynthesizable polymeric materials help to level the amount of petroleum-derived plastics currently being produced, which take at least 100 years to decompose. The group of biopolymers is very broad, and there are many types of biopolymers that match traditional plastics in their properties. After modification, these polymers often have significantly better properties than the original raw material or plastics [1,2,3].

**Figure 1:**
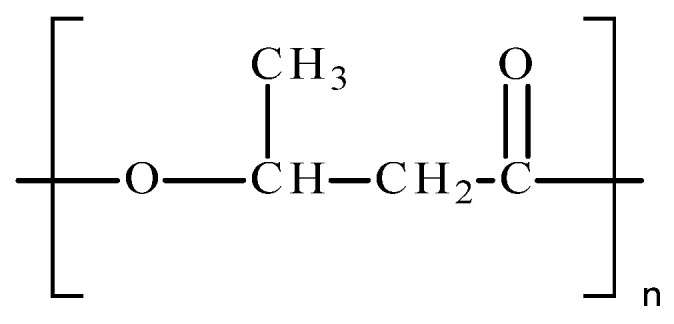
Structure of poly(3-hydroxybutyrate).

Poly(hydroxyalkanoates) (PHAs) are natural biopolymers belonging to the biopolyester and the aliphatic polyester groups. They are used for the intracellular storage of excess carbon and energy by microorganisms [4,5,6,7,8]. Poly(3-hydroxybutyrate) (P3HB) is one of the most widespread, widely studied and deeply described microbial biopolymers belonging to the PHA family [9,10]. It is produced and stored by some bacteria during times when the microorganism has insufficient food, and when unfavorable environmental conditions arise [11,12,13]. This compound is the most commonly synthesized PHA. When it is separated from cells, P3HB has a crystalline structure, and owes its remarkable crystallinity to its linear chain structure. P3HB belongs to the group of semicrystalline polymers with two phases, crystalline and amorphous, with their degree of crystallinity reaching up to 80% [14,15]. Its structure (Figure 1) is characterized by the presence of an ester functional group and a methyl side substituent that is responsible for some of the properties of P3HB.

This biopolymer can be produced from sugars, vegetable oils and fatty acids [11,16]. Its production is also possible as a result of continuous bacterial fermentation processes and various varieties of batch culture [17,18].

P3HB has many similar properties to polypropylene and PHAs, such as non-toxicity, UV resistance, biocompatibility, hydrophobicity, and thermoplasticity. In contrast, its own properties, different from PHAs, can include stability under harsh conditions, high oxygen impermeability and biodegradability under almost any environmental conditions [19,20]. The compound also has high barrier properties, and is soluble in hydrocarbons with chlorine in their structure, including chloroform, while it is insoluble in water, and shows poor acid resistance. The disadvantages of P3HB include the difficulty of recovery and high cost of obtaining this polymer, as well as its brittleness, low deformability, low degradation temperature, and the release of an unpleasant odor during processing [21,22].

In order to modify the thermal, mechanical, and some of the functional properties of P3HB, it can be mixed with other polymers, such as other polyhydroxyalkanoates, polycaprolactone and natural polymers, e.g., alginate, cellulose and chitosan. In this way, polymer compositions that are still biodegradable are produced [23,24,25]. Mixing P3HB with chitosan causes a decrease in the degree of crystallinity of P3HB and a decrease in the melting and glass transition temperature. The addition of chitosan contributed to increasing the hydrophilic properties of the obtained material, and improved P3HB’s physico-mechanical properties [26,27].

Polyurethanes are rarely used to modify the properties of P3HB except for in our works [28]. The polyurethanes were used to modify the properties P3HB, and more often to modify copolymers P3HB, poly(3-hydroxybutyrate-co-3-hydroxyvalerate) (PHBV).

In turn, Olkhov et al. used P3HB to modify the properties of polyamide [29]. The resulting blends were characterized by biodegradability, higher thermal than P3HB and lower crystallinity degree. Moreover, the diffusion mobility of water increases exponentially with the increase in the content of hydrophilic polyamide in the system.

Poly(3-hydroxybutyrate-co-3-hydroxyvalerate)/thermoplastic polyurethane composites were prepared by solution blending combined with melt processing by Wang et al. The incorporation of thermoplastic polyurethane decreased the blend crystallinity and increased the initial degradation temperature by 5 °C, and the elongation-at-break was increased by 225% compared with PHBV [30].

Martínez-Abad et al. described that the presence of thermoplastic polyurethane did not affect the crystallinity of the PHBV. They observed a gradual decrease in the modulus of elasticity and tensile strength with increasing polyurethane content in the blends. The barrier properties and the biodisintegratibility of PHBV were maintained [31].

Panaitescu et al. [32] modified the properties of PHBV with two polyurethane elastomers containing poly(epsilon-caprolactone) and poly(epsilon-caprolactone)/poly(butylene adipate) as the soft segment and the hard segment. The addition of 5–15 wt. % PU elastomers did not significantly influence the thermal stability of these blends, but decreased the crystallization temperature of PHBV (up to 9 °C) and the crystallinity degree by about 4–5%. The tensile strength and modulus of blends remained close to those of neat PHBV, but elongation at break increased up to 70%.

Frone et al. [33] modified P3HB with microfibrillated cellulose and a thermoplastic polyurethane containing biodegradable segments (described in [32]) via two routes—using a master batch and by direct mixing. The modifiers improved the thermal stability of PH3B (13 °C) and slightly decreased its melt viscosity, improving the melt processability. PU modifiers delayed the degradation of the P3HB composites.

There have also been studies wherein P3HB was used to modify the properties of polyurethanes. Saha et al. [34] synthesized PUs from castor oil and poly(3-hydroxybutyrate)diol using hexamethylene diisocyanate as a crosslinking agent. The presence of P3HB significantly increased the tensile strength, stiffness and crystallinity of the resulting PUs compared to neat CO-based PU.

In this study, in order to modify the properties of P3HB, polymer blends were produced with the use of a linear polyurethane based on diphenylmethane 4,4′-diisocyanate and polypropylene glycol with a molar mass of 400 g. The polyurethane was dosed at 5, 10 and 15 wt. % relative to the polymer blends. The effects of the presence and amount of polyurethane on the miscibility of the components, morphology, surface roughness, and thermal and mechanical properties of the polymer blends were investigated.

## 2. Materials and Methods

### 2.1. Materials

P3HB was supplied by Biomer (Krailling, Germany); its average molecular weight was M_w_ = 443,900 g-mol^−1^ and its dispersity index was (M_(w)_·M_n_^−1^) = 5.72; the P3HB melt flow index was 0.11 at 180 °C and 2.16 kg. 4,4′-diphenylmethane diisocyanate (MDI) and dibutyltin dilaurate (DBTL) were supplied by Sigma-Aldrich (Saint Louis, MO, USA), the 400 g molar weight polypropylene glycol (PPG400) by Sigma-Aldrich and the acetone by Chemsolute (Renningen, Germany).

#### Synthesis of Linear Polyurethanes

**Figure 2:**
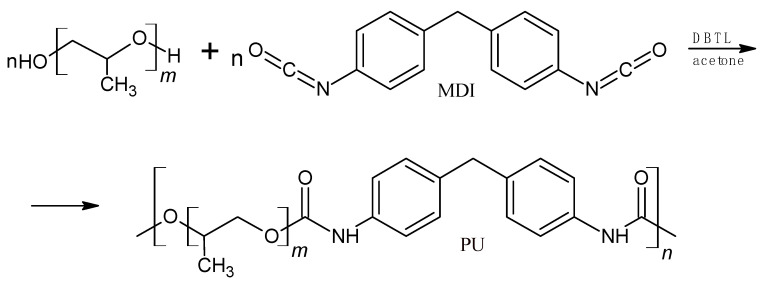
Synthesis scheme of polyurethane, where m ≈ 7, n ≈ 27.

The synthesis of polyurethane (Figure 2) was conducted in nitrogen. The molar ratio of isocyanate groups to hydroxyl groups of glycol was 1:1.08. The catalyst was used at the amount of 0.003 mol/mol PPG400. The synthesis procedure is described in [28].

### 2.2. Methods

#### 2.2.1. Manufacturing Technology of Polymer Blends

P3HB–PU blends were extruded using a ZAMAK RES-2P12A Explorer twin-screw extruder (ZAMAK Mercator company, Skawina, Poland). Polymer blends containing P3HB and a modifier, in the form of a linear polyurethane, were produced using PU in amounts of 5, 10 and 15 wt. %, as shown in Table 1.

The PU was dissolved in acetone, and the solution was homogenized with P3HB by mixing in a drum mixer (ERY Food Machinery, Warsaw, Poland). The homogenization process lasted 20 min and was carried out at room temperature. Then, the homogenized mixture was placed into a vacuum dryer to remove acetone. Finally, the mixture was fed into a co-rotating twin-screw extruder and the extrusion process was carried out under the conditions shown in Table 2. The speed of screws in the extrusion line was 40 rpm and the screw diameter was 12.5 mm, while the L·D^−1^ ratio was 24. After cooling in a cooling bath, the melt was pelleted and dried at 60 °C for more than 2 h.

#### 2.2.2. Mechanical Tests

A Zwick Z030 (ZwickRoell, Ulm, Germany) testing machine was utilized to assess the tensile properties of the prepared blends. The uniaxial tensile test was conducted according to EN ISO 527-1 [35] for molded specimens with a dumbbell shape. The specimens were produced using the following injection parameters:

- Injection speed—50 cm^3^/s;
- Mold temperature (Tf)—50 °C;
- Injection pressure (pd)—350–390 bar;
- Dwell time (Td)—30 s.

Each sample series comprised 7 specimens for subsequent statistical analysis. From the test results, Young’s modulus (E), tensile strength (σ_M_), and relative elongation at maximum tensile stress (ε_M_) were evaluated. The data were processed statistically, with the arithmetic mean (x), standard deviation (s), and coefficient of variation (V) being calculated.

**Figure 3:**
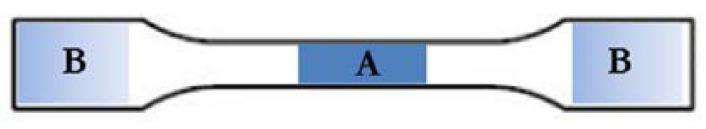
Zones of the hardness test specimen: A—measuring zone, B—gripping zone.

A Brinell hardness test of the blends was performed in accordance with EN ISO 2039-1 [36], specifically in zone A of the sample (Figure 3), intended for the uniaxial tensile test. Each specimen series contained seven samples, and statistical analysis was conducted.

An impact tensile test was also conducted on the blend samples. The impact tensile strength was determined in accordance with [37], using a CEAST 9050 pendulum hammer (Illinois Tool Works Inc., Glenview, IL, USA), with uniaxial tensile test paddles employed for the procedure. The specimen geometry was adjusted according to the standard, with notches milled for entire specimen batches. Each series consisted of 7 specimens, and statistical analysis was performed as well.

#### 2.2.3. Roughness Measurements

The samples were visually assessed using an Olympus DSX 1000 microscope (Olympus Corporation, Tokyo, Japan). The surface was assessed using a non-contact measurement method, which was necessary due to the softness of the material being tested. This is because the use of a stylus tip could lead to damage to the surface of the samples. Measurements were taken on a panoramic image composed of four images with a total area of 2891 µm × 2156 µm. The LEXT application dedicated to the DSX series was used for the analysis. The length of the measurement section was approximately 2900 µm. The roughness profiles were determined in accordance with [38].

A surface roughness profile is a line that describes microscopic irregularities on the surface of a material. It is the result of a roughness measurement, which assesses differences in height on the surface along a specific line. This profile provides information about surface properties such as smoothness, roughness or texture, which is important in many industrial and engineering applications.

Arithmetic mean roughness (R_a_): This is the most commonly used parameter that measures the average absolute value of the deviation of the profile from the centerline in a given section. R_a_ gives an overall assessment of surface roughness.

Profile maximum height (R_z_): This is the sum of the height of the highest peak and the deepest valley in a section. R_z_ is more sensitive to single high irregularities than R_a_.

Average peak height (R_p_): This is the average height of the peaks above the centerline.

Average depth of valleys (R_v_): This is the average depth of the valleys below the centerline.

Roughness profile width (R_sm_): This is the average distance between adjacent roughness peaks.

Root mean square deviation (R_q_): Defines the square root of the mean of the squared Z(x) values within the sampling length.

Mean height (R_c_): Represents the average value of the height of profile elements measured over the sampling length.

The interpretation of the roughness profile can be performed as follows:

- Smooth surface. Low R_a_, low R_z_—the surface is generally flat and even;
- Rough surface. High R_a_, high R_z_—the surface has many irregularities and a large variation in height;
- Characteristic profile. Specific values of R_p_, R_v_ and R_sm_ can indicate specific manufacturing features such as cutting, chemical treatment, abrasion, etc.

### 2.3. Analytical Methods

#### 2.3.1. HT-GPC Chromatography

To determine the molar mass of linear polyurethane, an HT-GPC 220 chromatograph (Agilent Technologies, Santa Clara, CA, USA) was used, which has a detection set-up that allows refractive index detection and viscosity detection. Samples were first dissolved in dimethylformamide (DMF) (Sigma-Aldrich, Saint Louis, MO, USA) and then separation and detection were performed at 50 °C using DMF. The flow rate was 1 mL/min and the injected sample volume was 100 µL. Polystyrene standards (12 standards) in the range 580 kDa–271 Da were selected to calibrate the GPC. The polyurethanes were characterized by determining Mn—number average molar mass, Mw—weight average molar mass, Mp—molar mass at peak maximum and DI—degree of dispersion. The results obtained can be found in Table 3.

#### 2.3.2. FTIR Spectra

In investigating the interactions of the blend components, FTIR spectra of the P3HB, PU, and P3HB-PU blend in ATR form were performed with the use of an ALPHA FT-IR spectrometer (Bruker, Shanghai, China) in the wave number range 400–4000 cm^−1^, with a resolution of 2 cm^−1^.

#### 2.3.3. Scanning Electron Microscopy

The morphology of the polymer blends obtained was studied using a JEOL type JSM-6490 LV (JEOL Ltd., Tokyo, Japan) scanning electron microscope with a dispersion analyzer of X-ray energy for micro-area chemical analysis. The samples were first subjected to freezing in liquid nitrogen, after which they were broken with a hammer and the fracture surfaces of the samples were examined. In order to obtain an SEM image, their surfaces were sprayed with a thin metallic layer, which prevents the accumulation of surface charges hindering or even preventing the observation of the surface. The samples prepared in this way were coated with a layer of gold about 10 mm thick using a JEOL JFC-1300 (JEOL Ltd., Tokyo, Japan) gold sputtering machine, resulting in microphotographs showing the morphology along with the surface structure.

#### 2.3.4. EDS

The distribution of elements in the polymer biocomposites was analyzed using an Energy Dispersive Spectroscopy (EDS) detector integrated into an Axia ChemisSEM Scanning Electron Microscope (SEM) from ThermoFisher Scientific (Waltham, MA, USA). The electron beam source used was a tungsten filament. Prior to analysis, the samples were sputter-coated with a 0.1 μm thick gold layer to enhance conductivity. The analysis was conducted at a magnification of 250× and a voltage of 30 kV.

#### 2.3.5. Thermogravimetric Analysis

Thermogravimetric (TG) analysis of P3HB and the resulting polymer blends was conducted using a Mettler Toledo AG—TGA/SDTA851e (Mettler-Toledo International Inc., Columbus, OH, USA) thermogravimetric analyzer. The samples were heated at a rate of 5 °C per minute over a temperature range of 25 °C to 400 °C. Measurements were performed in both nitrogen and air atmospheres. The following parameters were determined:

- Temperature at the onset of decomposition (Ton);
- Half-mass loss temperature (T_50%_);
- Degradation temperatures at 10% and 5% mass loss (T_10%_, T_5%_);
- Temperature of the fastest decomposition (Tmax);
- Residue at 400 °C.

#### 2.3.6. Differential Scanning Calorimetry

The temperature dependence of the heat flow rate was determined using a differential scanning calorimeter (DSC822e, Mettler Toledo, Columbus, OH, USA). All analyses were conducted under a nitrogen atmosphere with a constant flow rate of approximately 50 mL·min^−1^. The experiments were performed over a temperature range from −90 °C to 195 °C. The accuracy of the specific heat measurements was approximately 3%. The measurements were conducted at a cooling rate of 10 °C·min^−1^ and a heating rate of 10 °C·min^−1^. The first heating cycle was performed to erase the thermal history of the samples, while the second heating cycle was used to evaluate the properties of the samples. The results were compared based on the second heating run.

#### 2.3.7. Water Contact Angle (WCA)

An OCA 15 optical goniometer equipped with a module for the automatic dosing of measuring drops from Data Physics was used, according to the PN-EN 828 standard [39]. The contact angle is the angle formed by the tangent to the surface of the measuring drop deposited on the surface of a solid, at the point of contact between the three phases of solid, liquid, and gas. Measurements were made at room temperature. The drop contours were made using the SCA20U computer software Version 6.1 and the contact angle was calculated. Ten measurements were taken for each blend tested, and the arithmetic mean of all measurements was taken as the final result. The mean values of contact angles are shown with a 95% confidence limit.

## 3. Results and Discussion

The disadvantages of ‘double-green’ P3HB are its brittleness and low thermal stability, and therefore, its difficult processing conditions. In the present study, a linear polyurethane obtained by reacting polypropylene glycol with a molar mass of 400 g with MDI (Figure 2) was used to improve the properties of P3HB. The molar mass of M_w_ PU is 17,588 g (Table 3). To modify the properties of P3HB, PU was used at 5, 10 and 15 wt. % (Table 1). The prepared polymer blends were melt-mixed using a twin-screw extruder. P3HB was processed in a similar manner. In sequence, the miscibility, structure, roughness and surface morphology of the polymer blends were investigated.

### 3.1. Blend Characterization by FTIR Spectroscopy

**Figure 4:**
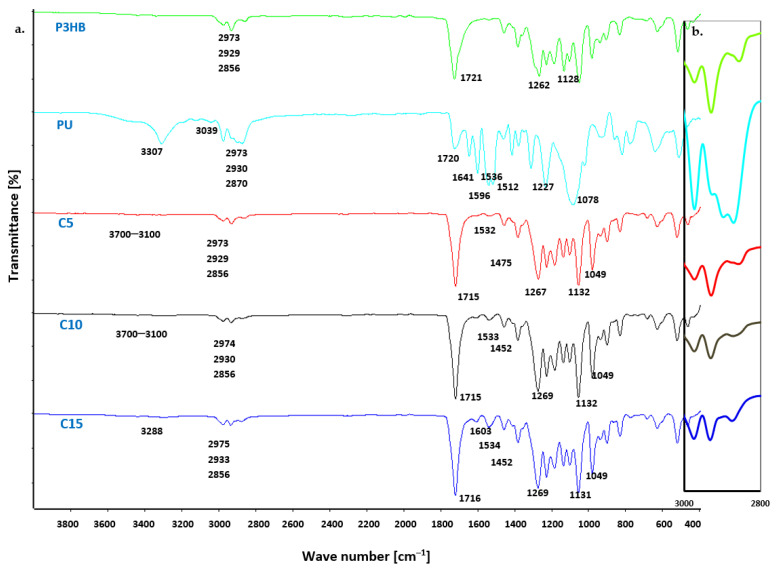
(**a**) FTIR spectra of P3HB, PU and P3HB-PU blends containing 5, 10 and 15 wt. % PU (designated C5, C10 and C15) and (**b**) magnified image of the region 2800–3000 cm^−1^.

FTIR characterization was performed to investigate the possible interactions and compatibility between the components of the blends [40]. The FTIR spectra of the prepared P3HB-PU blends were compared with the individual FTIR spectra of P3HB and PU. The observed differences and similarities in the bands of the polymer blends are presented in Figure 4. The characteristic bands of virgin P3HB were observed, confirming the structure of the blend’s matrix. Notably, a strong ester bond band, originating from the valence vibrations of the C=O bonds, appeared at 1721 cm^−1^. Additionally, asymmetric and symmetric C–O bond vibration bands of the ester group were identified at 1262 cm^−1^ and 1129 cm^−1^, respectively. Furthermore, asymmetric and symmetric C–H bond vibration bands of the methyl and methylene groups were found at 2973, 2929, and 2856 cm^−1^ [41,42].

In the FTIR spectrum of PU, a valence vibration band of the N–H bond from the urethane group (-NH-COO-) was observed in the 3600–3100 cm^−1^ range. This broad and fuzzy band indicates the presence of hydrogen bonds in the polyurethane. The C–H bonds of PU methylene groups exhibited a single broad band at 2973, 2930, and 2870 cm^−1^, while a C–H bond vibration band of the phenyl ring appeared at around 3039 cm^−1^. The C=O bond vibrations of the -NH-COO- urethane group were observed at 1720 cm^−1^, and a characteristic deformation vibration band of the N–H bond of polyurethane appeared at 1596 cm^−1^. Additionally, characteristic backbone bands of the aromatic ring were visible at 1641, 1536, 1512, and 1475 cm^−1^. Bands of asymmetric and symmetric C–O and C–N bonds of the -NH-COO- groups were visible at 1227 and 1078 cm^−1^ [43]. FTIR analysis of the PU confirmed the correct progression of the polyaddition reaction during PU synthesis. The spectra of the blends containing varying amounts of PU showed both similarities and differences (Figure 4). These spectra also differed in terms of peak intensity and range when compared to the peaks present in the spectrum of the pure polyester (P3HB) and the PU modifier alone.

In the FTIR spectra of all P3HB-PU blends (Figure 4), a low-intensity band was observed in the range of 3700–3100 cm^−1^. This band’s intensity increased with the increasing PU content in the blends. In contrast, a diffuse band in the 2800–3000 cm^−1^ range showed variable intensity, depending on the amount of PU present. In the blend containing 10 wt. % PU, this band was much smaller compared to those of pure PU or P3HB. However, the intensity of the band was comparable in samples containing 5 wt. % and 15 wt. % PU.

Additionally, a band at 1715 cm^−1^ appeared in all blend spectra, which was attributed to the stretching vibrations of the C=O bonds from the P3HB carbonyl group and the -NH-COO- group of the PU modifier. The carbonyl group band was shifted to lower wavenumber values, from 1721 to 715 cm^−1^. The observed shift can be attributed to hydrogen bonds; therefore, all C=O groups of P3HB are apparently connected by hydrogen bonds, since neither component can be observed at higher wavenumbers attributed to free C=O groups [44]. Moreover, it should be noted that the lowest intensity of this band was shown by the blend containing 5 wt. % PU (blend C5), which indicates the presence of carbonyl group interactions in the components. There was a bending vibration band of N–H urethane bonds, at a wave number of 1603 cm^−1^, which was characterized by low intensity due to the formation of intermolecular hydrogen bonds between the P3HB and PU [45,46].

The intensity of the band at 1715 cm^−1^ was variable and dependent on the amount of PU in the polymer system, with the highest intensity observed for the sample containing 15 wt. % PU. This suggests that specific interactions were most prominent in the sample modified with 15 wt. % PU.

Additional characteristic bands were identified and analyzed. Typical asymmetric and symmetric C–O vibrational bands of esters and urethanes appeared at wave numbers of 1269 cm^−1^, 1132 cm^−1^, and 1049 cm^−1^. FTIR spectral analysis of the prepared polymer blends and their individual components confirmed the interaction between PU and the P3HB matrix. This indicates that the PU modifier successfully interacted with the P3HB, influencing the properties of the resulting blends.

### 3.2. Analysis of Topography and Roughness of Manufactured Polymer Blends

**Figure 5:**
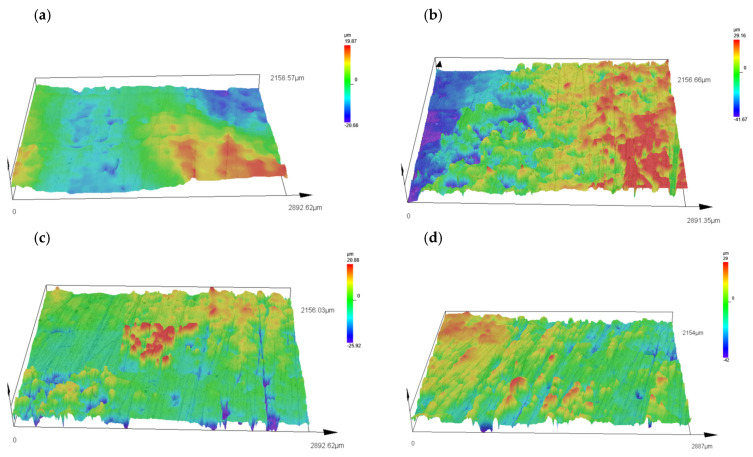
Surface topography of samples with (**a**) P3HB; (**b**) C5; (**c**) C10 and (**d**) C15.

Figure 5 shows the surface topographies of the samples, namely, native P3HB and the polymer blends with PU added at 5, 10 and 15 wt. %. The analysis of the topographies showed that the surface of the P3HB sample is relatively flat, suggesting minimal surface disruption. For the C5, C10 and C15 samples with 5%, 10% and 15 wt. % PU, there is a noticeable change in the surface topography, characterized by a higher proportion of protuberances, which are particularly pronounced on sample C5 with the addition of 5 wt. % PU (Figure 5).

The surface of sample C5 shows a significant amount of protrusion, indicating a significant change in surface topography. These protuberances are clearly visible and suggest an intense disruption of the surface structure compared to sample P3HB. The change in topography of sample C10 is still noticeable, but appears to be less intense compared to sample P3HB. The surface shows moderate disturbance, suggesting some stabilization of the roughness structure. The surface of sample C15 shows similar changes in topography as in sample C5.

The surface of sample P3HB (Figure 5a) is relatively smooth, with a small roughness range of ~−20 to ~20 µm. This suggests minimal structural disturbances and a homogeneous surface. The addition of 5 wt. % PU (sample C5, Figure 5b) leads to a significant increase in roughness, with a roughness range of ~−40 to ~30 µm. Prominent prominences and depressions are observed, indicating intense surface disturbances. This may be due to the inhomogeneous distribution of PU in the P3HB matrix, which leads to local structural stresses. In sample C10 (Figure 5c), containing 10 wt. % PU, the surface topography shows some smoothing compared to C5. The roughness is in the range of ~20 to −26 µm, suggesting the partial stabilization of the structure. This may be due to the better compatibility of P3HB and PU at this modifier content, which reduces the occurrence of sharp surface irregularities. Sample C15 (Figure 5d), containing 15 wt. % PU, again shows a larger range of irregularities (~−40 to ~30 µm), but the surface structure seems more regular and homogeneous compared to C5. This may suggest that the higher PU content leads to a more even distribution of the polyurethane phase, which reduces local stresses and results in a more coherent surface structure.

Visual examination with an Olympus DSX 1000 microscope allowed a detailed analysis of the surface topography of the samples. The results suggest that the modifying additive significantly affects the surface structure, particularly at 5 wt. % PU additive, where the greatest changes are observed. The non-contact measurement method proved to be suitable for this type of material, allowing an accurate assessment of the topography without the risk of surface damage. Further analysis can focus on the impacts of these topographical changes on the mechanical and performance properties of the samples tested.

**Figure 6:**
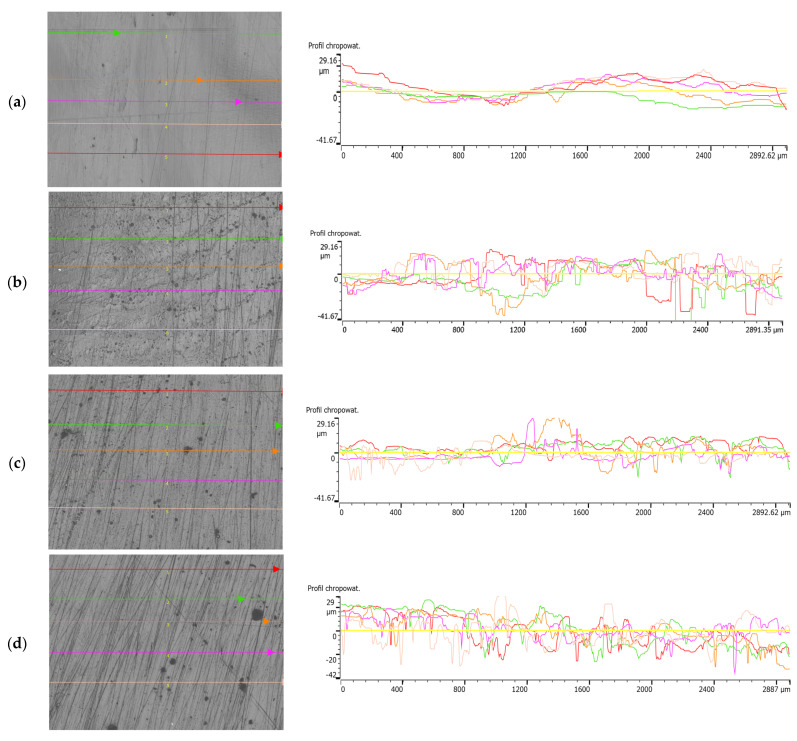
Surface of samples: (**a**) P3HB, (**b**) C5, (**c**) C10 and (**d**) C15, with lines marking the roughness measurement locations and roughness profiles.

The surface roughness of the specimens was measured in five designated directions, as shown in the photographs of the specimens. The results of the measurements are illustrated in Figure 6, which show the roughness profiles.

Table 4 shows a summary of the surface roughness parameters for P3HB and its blends using different percentages of PU content—5%, 10%, 15 wt. %. Parameters such as R_p_, R_v_, R_z_, R_c_, R_a_, R_q_ and R_sm_ are tabulated, and their average values for each group are calculated. The P3HB samples show relatively low values for the roughness parameters. These results serve as a benchmark for comparison with the modified samples. The values of R_a_ and R_q_ suggest that the surface is relatively smooth, and the high value of R_sm_ indicates a large distance between roughness peaks. In the case of C5 samples containing 5 wt. % PU, we observed a significant increase in all roughness parameters. The increases in R_v_ and R_z_ are particularly pronounced, which indicate greater valley depths and higher overall roughness heights. This is significant evidence of the significant modifying effect of 5 wt. % PU on the surface structure. When P3HB is modified with 10 wt. % PU, the values of the roughness parameters are higher than for the unmodified samples, but lower than for the C5 samples. These increases are particularly evident for the parameters R_p_, R_v_ and R_z_, suggesting a moderate increase in surface roughness. For the modification of P3HB with 15 wt. % PU, the results show a further increase in roughness compared to the P3HB and C10 samples, but they do not reach the extreme level as with 5 wt. % PU. The mean distance between peaks (R_sm_R_sm_R_sm_) is the lowest in this group, suggesting a more regular and dense roughness structure.

The most significant increase in roughness parameters was observed when P3HB was modified with 5 wt. % PU. This suggests that this level of modification has the strongest effect on the surface structure. At 10% and 15 wt. % PU, the polymer blend samples show higher parameter values than for unmodified samples, but the increase in roughness is not as drastic as for C5.

An increase in the proportion of PU in the polymer blend results in an increase in surface roughness parameters, but this is not a linear increase. The values of R_v_ and R_z_ are particularly sensitive to changes, which can be crucial in applications requiring specific surface properties. The R_sm_ value decreases with increasing amounts of polyurethane modifier, indicating a more regular and dense roughness structure at higher modification levels.

Further research could focus on the effects of these parameters on the functional properties of the materials, such as abrasion resistance, adhesion or surface chemical reactions, allowing a more complete understanding of the effects of the modifications on the functionality of the materials.

### 3.3. Analysis of Contact Angle and Surface Roughness of Polyurethanes with PU Additive

As part of the conducted research, the surface topography of natural P3HB and polymer blends with the addition of polyurethane (PU) in amounts of 5%, 10%, and 15% by weight was analyzed. The aim of the study was to determine the effects of PU addition on the surface structure of the materials, and to examine how roughness changes depending on the concentration of the additive. Simultaneously, contact angle analysis was performed to assess the influence of the microstructure on surface wettability.

The P3HB sample exhibited an average contact angle of 72.14° (95% CI), indicating its hydrophobic nature. The surface structure of this material was relatively smooth and homogeneous (Table 5), as confirmed by the roughness analysis performed using the Olympus DSX 1000 microscope. The absence of distinct protrusions and depressions suggests a stable chemical structure, likely due to the regular distribution of ester bonds in the P3HB polymer. The homogeneous structure of P3HB limits liquid adsorption, contributing to the high stability of wettability.

The addition of 5% PU caused a significant increase in surface roughness, visible as distinct irregularities and protrusions. The average contact angle was 54.13° (95% CI), suggesting increased hydrophilicity compared to P3HB. The pronounced irregularities in surface topography may result from non-uniform PU dispersion, leading to local variations in surface energy distribution (Table 5). The chemical properties of PU, including the presence of carbonyl and hydroxyl groups, may have promoted interactions with water, further enhancing wettability.

For the C10 sample, the average contact angle was 58.17° (95% CI). The surface roughness was still considerable, but exhibited a more balanced pattern compared to the C5 sample. The increase in PU concentration led to the partial homogenization of the surface, possibly due to better phase compatibility between PU and P3HB. The stabilization of the contact angle can be explained by an increased number of hydrogen-bonding interactions between PU and P3HB, reducing the contrast between hydrophilic and hydrophobic surface areas.

The highest PU content (15%) resulted in a further increase in the contact angle to 62.23° (95% CI). Topography analysis showed greater surface uniformity, with fewer distinct protrusions and depressions than in the C5 and C10 samples (Table 5). The improved PU dispersion may result from a higher number of intermolecular interactions, such as hydrogen bonds between urethane segments, stabilizing the surface structure. The increase in surface hydrophobicity at higher PU concentrations may be due to a greater presence of hydrophobic PU segments, reducing water contact with the surface.

The relationship between surface roughness and the contact angle indicates that microstructural irregularities in the C5 sample led to a decrease in the contact angle, which can be explained by a local increase in surface energy due to PU aggregation. In contrast, a higher PU content (C10 and C15) stabilized the surface, reducing its heterogeneity and leading to a moderate increase in hydrophobicity.

Chemically, this effect can be explained by changes in the hydrogen bonding network and differences in the distribution of hydrophilic and hydrophobic segments. At lower PU concentrations (C5), hydrophilic interactions dominated due to the presence of polar carbonyl and urethane groups. At higher PU concentrations (C15), the increased number of hydrophobic segments (from PU) led to greater hydrophobicity and a more stable surface.

These findings are particularly relevant for applications in biomedical engineering and protective coatings, where controlling surface wettability is crucial for material interactions with the environment.

### 3.4. Mechanical Properties of Polymer Blends

**Figure 7:**
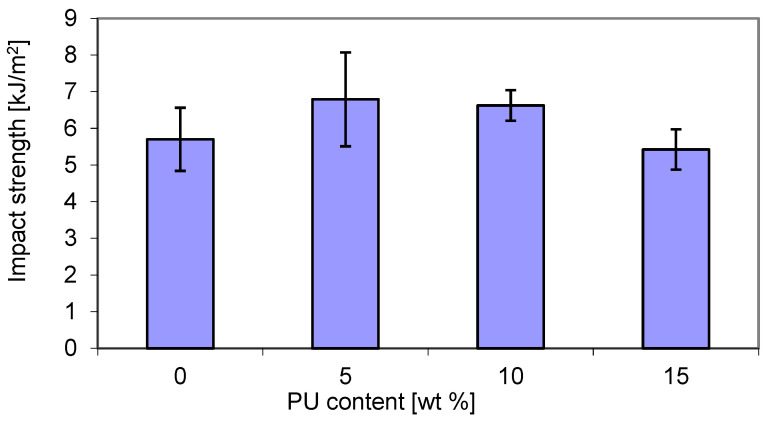
Effect of polyurethane (PU) content on the impact tensile strength of P3HB–PU blends.

The results of the mechanical tests of the obtained P3HB–PU blends are shown in Figure 7 shows the impact tensile strength (IS) of P3HB samples as a function of polyurethane (PU) content. It can be noted that the impact strength (IS) increased with increasing PU content. The maximum IS value was exhibited by the blend containing 5 wt. % PU and was higher by approximately 20% than that of the virgin P3HB matrix. The decrease in IS at a higher PU content can be attributed to the absence of interactions between the P3HB and PU, but also the saturation of the modifier leading to phase separation and a decrease in the impact strength [47]. However, the lowest IS value was shown by the blend based on 15 wt. % PU, and was comparable to that of the P3HB matrix.

The improvement in impact resistance of P3HB/PU blends can be attributed to the formation of interpenetrating polymer networks with good homogeneity and increased reinforcement. An improvement in the tensile strength of the P3HB blend with 5 wt. % PU has been reported in another study [48]. Feng et al. observed the enhancement of impact strength in blends of polylactide and polyurethane [49].

**Figure 8:**
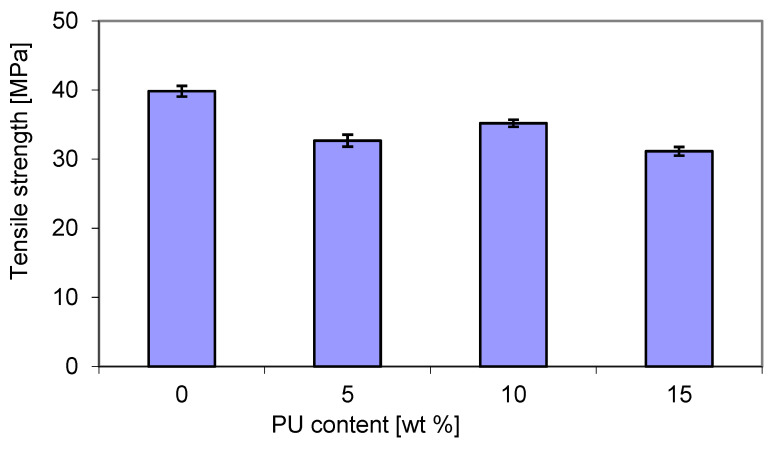
Tensile strength of P3HB–PU blends as a function of PU content.

However, as shown in Figure 8, the tensile strength (TS) exhibited a slightly different trend in connection with PU content. The addition of PU led to a decrease in the TS of blends due to the presence of flexible PU chains. It turns out that the highest TS (over 35 MPa) was recorded for blends containing 10 wt. % PU. However, an increase in the PU content to 15 wt. % caused a decrease in the TS of about 31 MPa. An intermediate value of about 32.5 MPa was recorded with 5 wt. % PU. The tensile strength of pure P3HB was approx. 13% higher than that of the latter blend. Wang et al. [50] showed that the tensile strength and elastic modulus blends of polylactide–thermoplastic polyurethane were sensitive to the volume fraction of polyurethane. Polylactide played a dominant role in the tensile strengths of those composites. It is well known that the miscibility and the compatibility between the blend components, i.e., poly(3-hydroxybutyrate-co-4-hydroxybutyrate) and thermoplastic polyurethane, have a significant effect not only on the tensile strength, but also on other performance properties and blend processability [51]. However, it seems that the compatibility between poly(3-hydroxybutyrate) and polyurethane appears to be limited, but could be increased. It has been reported that the crystallization temperature and crystallinity of PHBV decreased due to the presence of PU, leading to some specific interactions between the two components [32].

**Figure 9:**
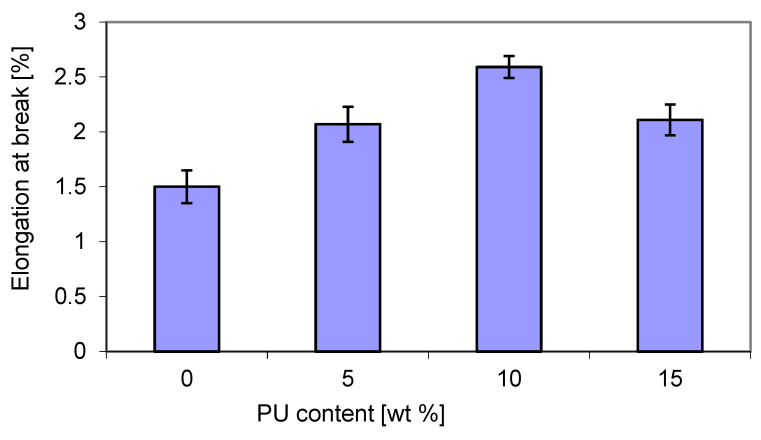
Effect of PU content on the relative elongation at breaks of P3HB–PU blends.

The relative elongation at break (Figure 9) showed an opposite trend to tensile strength (Figure 8). The maximum value was also obtained for 10 wt. % PU, and the lowest value for both 5% and 15 wt. % PU content. The elongation at break for the blends was higher than that of virgin P3HB. The presence of the greatest content of PU in a blend with P3HB caused a decrease in elongation at break, similarly to polylactide/polyurethane blends, as reported by Feng et al. [49].

**Figure 10:**
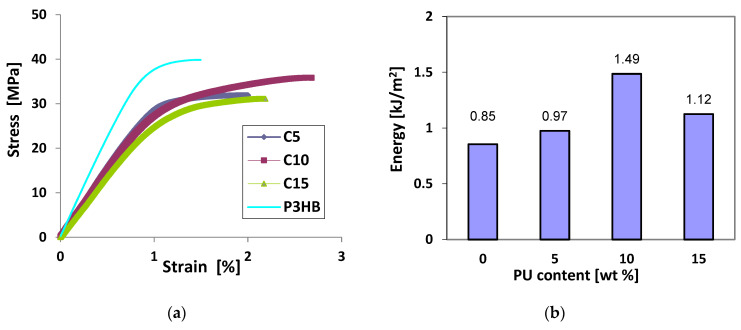
Stress–strain curves of P3HB containing polyurethane content (**a**) and energy to break of P3HB/PU (**b**) as a function of polyurethane content.

As shown in Figure 10, the stress–strain curves (a) of P3HB containing 5–15 wt. % polyurethane (designated C5-C15) were lower than those of the virgin matrix (P3HB). Furthermore, it can be noted that the slopes of the curves from 0 are also lower for the P3HB/PU blends than for the virgin matrix, confirming the increase in flexibility and ductility of the former. The energy to break of the blends (b), as determined by the areas under the different curves, increased for all blends containing polyurethane. A maximum improvement in fracture energy of ~75% compared to the virgin matrix was obtained with the blend prepared with 10 wt. % polyurethane. The increase in elongation at break and energy at break can be attributed to the existence of continuous and dispersed phases and interfacial adhesion [31]. However, Wang et al. [30] explained the significant increase in the elongation at break of the polyester matrix via the ductility and good toughness of polyurethane. Similar results were reported elsewhere [31].

**Figure 11:**
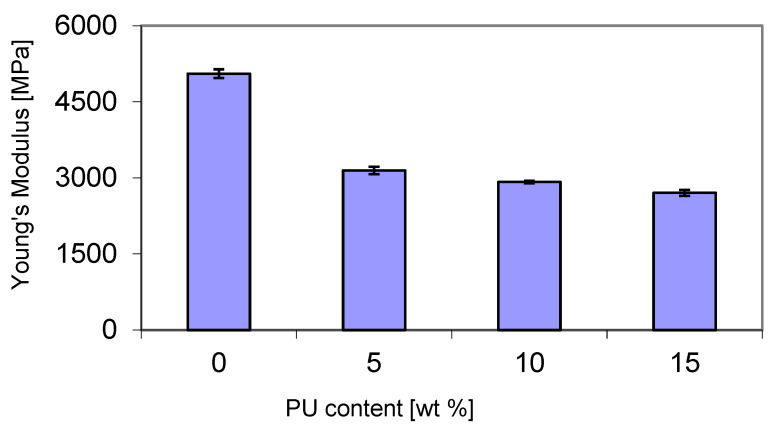
Effect of PU content on the Young’s modulus of the P3HB–PU blends.

A decrease in Young’s modulus was observed with increasing PU content in the P3HB matrix (Figure 11). Its value decreased from about 3150 MPa at 5 wt. % PU to about 2700 MPa at 15 wt. % PU. The significant reduction in Young’s modulus of about 40% for the blend containing 5 wt. % PU compared to pure P3HB can be attributed to the increase in compositional flexibility induced by the polymer modifier chains. Additionally, an increase in the free volume in the blend can also explain the decrease in the modulus and, with it, the stiffness of the blends.

**Figure 12:**
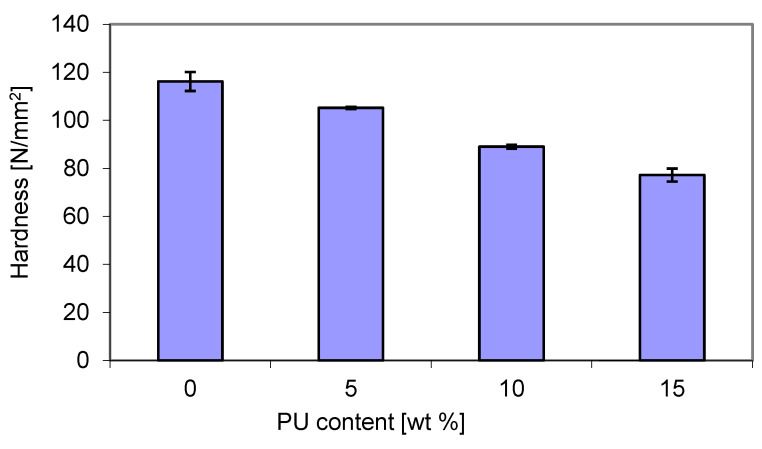
Hardness of P3HB–PU blends as a function of PU content.

The hardness tests were carried out using the ball press method, and the results are presented in Figure 12. As expected, and following the trend of Young’s modulus (Figure 10), the hardness decreased with increasing PU content, reaching the lowest value of 78 N/mm^2^ with a modifier content of 15 wt. %. This can be attributed to the increase in flexibility of the blends. The reduction in blend hardness can be related to the flexible polymer chains.

**Figure 13:**
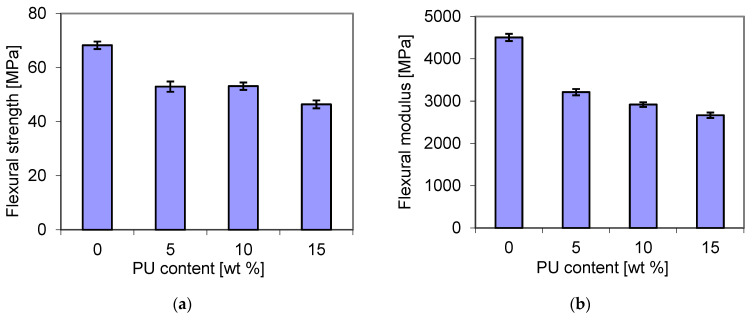
Dependence of the flexural strength (**a**) and flexural modulus (**b**) of the P3HB–PU blends on PU content.

In this study, bending tests were also carried out for the blends containing different amounts of polyurethane. As shown in Figure 13, the flexural strength (a) showed a similar trend to that of the flexural modulus, but it was different from that of the tensile strength (Figure 8). It decreased from 67 MPa for the pure matrix to about 53 MPa and 46 MPa for the blends that contained 10 wt. % PU and 15 wt. % PU, respectively. The reduction in some mechanically resistant properties has been reported for P3HB/PCL [52] and P3HB/PLA [53] blends. For example, the decrease in tensile strength was attributed to the formation of two phases with poor miscibility [53].

### 3.5. Morphology and Element Composition of Polymer Blends

**Figure 14:**
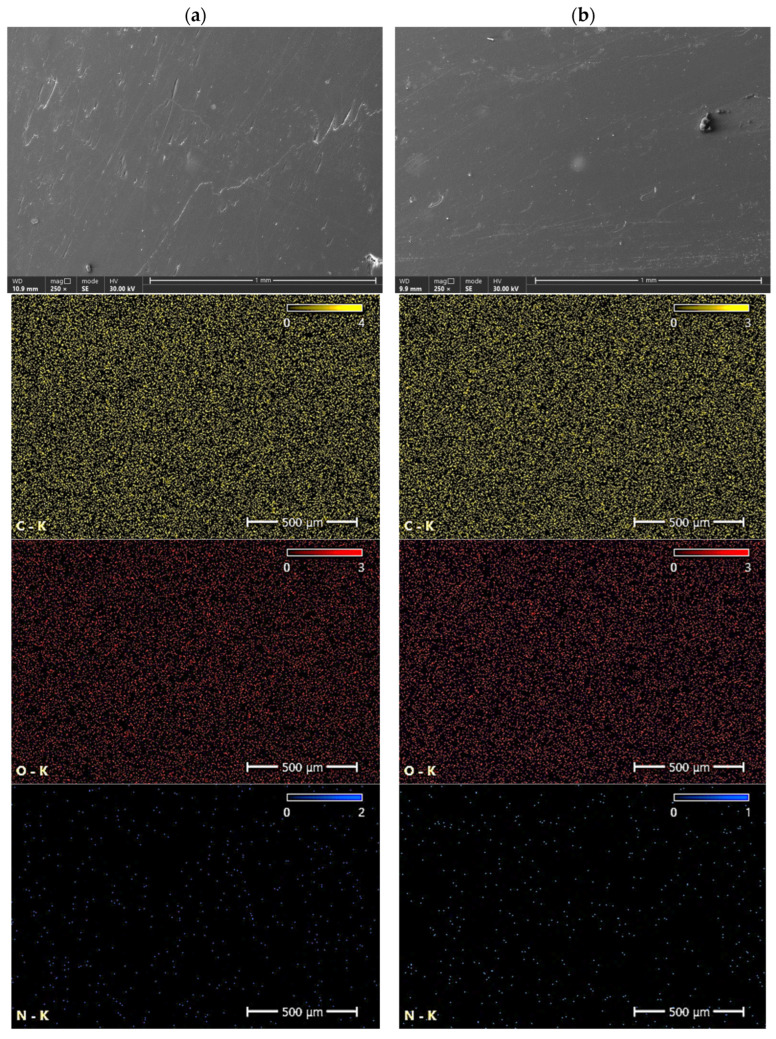
Microstructure with elemental maps of the samples: (**a**) P3HB, (**b**) C5.

Figure 14 shows surface microstructures of P3HB and C5, together with elemental maps. The determination of the chemical composition of the surfaces of the polymer variants studied was carried out by energy dispersive X-ray scattering (EDS). The yellow, red, and blue dots represent the detected carbon, oxygen, and nitrogen elements, respectively, while the black area indicates the absence of a specific element. Table 6 shows the results of the elemental analysis of the P3HB, C5, C10 and C15 polymer blends. Based on the analysis of the data obtained from the EDS tests and summarized in Table 5, slight changes in the chemical composition of the analyzed samples are evident. Correspondingly, for nitrogen, there is an increase in weight content from 5% in the P3HB sample to 5.6% at 15 wt. % PU (C15). The carbon content changes from a value of 51.0% (P3HB) to 43.5 (C5), 43.2% (C10), and 42.4% (C15). The oxygen content, on the other hand, decreases from 44.0% (P3HB) to 43.5% (C5), 43.2% (C10) and 42.4% (C15). An increasing trend can be observed as a polynomial function of the nitrogen content of the samples analyzed. In the case of carbon, there is a linear increase in the elemental content, while in the case of oxygen, there is a linear decrease in the elemental content by mass.

**Figure 15:**
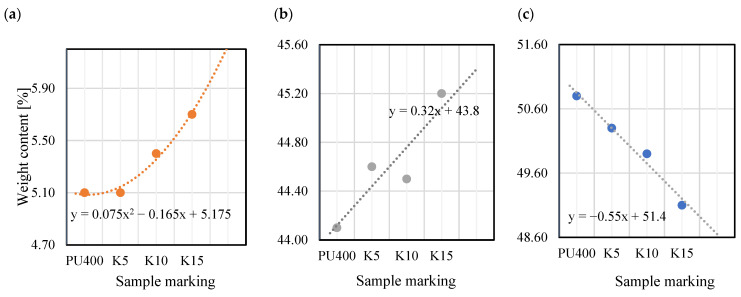
Weight content of elements as a function of increasing hardener content: (**a**) nitrogen; (**b**) carbon; (**c**) oxygen.

In Figure 15, for better visualization, the weight contents of the elements are presented as a function of the increasing content of the PU modifier. Although EDS is a semi-quantitative characterization tool, and its spectrum merely provides a measure of specific elements in the selected area [54], the present results indicate that with an increasing amount of modifier, the concentrations of nitrogen and carbon also increase (Figure 15).

**Figure 16:**
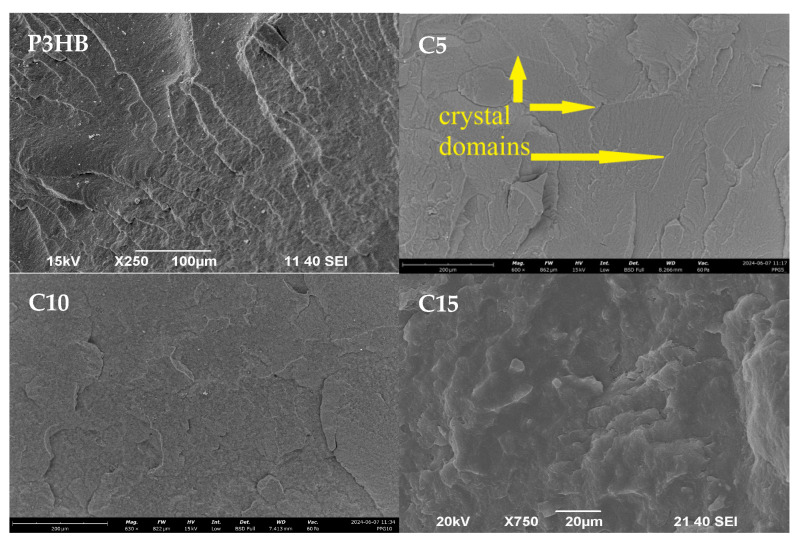
SEM photomicrographs of P3HB and its blends with PU in the amount of 5, 10, 15 wt. %, indicated as C5, C10 and C15, respectively.

Figure 16 shows SEM micrographs of the fracture surfaces of P3HB containing different amounts of PU (5 wt. %, 10 wt. % or 15 wt. %, designated C5, C10 and C15, respectively) based on MDI and short-chain polypropylene diol with a molecular weight of 400 g/mol. The images of the samples have been used to explain the mechanisms of interaction of the polyurethane, thus determining the mechanical properties of the tested blends. 

As shown in Figure 15, the fracture surface of the native P3HB was slightly wavy and glassy, suggesting the presence of a regular crack propagation path. P3HB itself has a semi-crystalline structure, which is related to the structure of the macromolecule. The areas of brittle fracture visible in the image are arranged almost unidirectionally. The introduction of the polyurethane modifier causes an apparent disruption in the continuity of the P3HB matrix structure, which disturbs the crystallization of P3HB. We note the rough, unidirectionally aligned crystalline domains can be seen on all micrographs of blends containing polyurethane. Crystallization between PU macromolecules is hindered for spherical reasons. PU has two large aromatic rings separated by a methylene group. The domains noticeable in the photographs cannot come from PU itself or P3HB itself, because they are not present in the micrograph of native P3HB. This indicates an interaction of the biopolymer matrix with the polymeric modifier, resulting in the spread of the interacting P3HB chains and the formation of rough domains preceded by wavy areas of the matrix. The linear, spatially small P3HB chain interacts, via hydrogen bonds of the C=O carbonyl groups, with the N–H groups from PU. This results in the formation of ordered adducts between P3HB and PU. The glassy interleavings characteristic of P3HB are thus separated by the crystalline regions of the adducts formed. The increased amount of PU projects their uneven distribution in the matrix, with a high probability of producing additional ordered domains between the chains of the modifier itself. Noticeable disentanglement of the glassy breakthroughs characteristic of P3HB is observed, and they are separated by PU-derived crystalline areas with wavy structure fragments. Their presence explains the elasticizing effect of PU. This explains the reduction in hardness and increase in strength of PU-modified blends. However, the introduction of 5 wt. % and 10 wt. % PU (C5 and C10) results in a uniform disruption of the continuity of the P3HB matrix, and a higher amount of PU (15 wt. %) implies the aforementioned occurrence of unevenly distributed rough areas in the presented breakthrough blend. This may explain the improvement in the discussed mechanical properties of the blends containing 5 wt. % (C5) and 10 wt. % (C10) PU, and the worsening of the properties of the blend containing the highest amount (i.e., 15 wt. % (C15)) of the PU modifier. In the case of the blend containing the highest amount of PU, we see the unfavorable agglomeration of the PU, and the rough areas are arranged differently and unevenly. This may be related to the uneven absorption of energy by this sample when a destructive force is applied during the test, which determines the worse properties of the samples compared to those containing 5% and 10 wt. % PU. Advantageously, the most uniform disruption of the continuity of the P3HB structure in the form of crystalline domains can be seen on the micrograph of the sample containing 10 wt. % PU, which explains the best properties of this blend.

### 3.6. Thermal Properties of Polymer Blends

In order to check the thermal stability of the resulted blends, thermogravimetric analysis was performed. The interpretations of obtained results are presented in Table 7. The TG analysis of the blends shows a thermal stability of the blends that is higher than that of the native P3HB. 

The temperature at the beginning of the decomposition (T_on_) of the blends containing 5 and 10 wt. % PU was almost 30 °C higher than that of pure P3HB, while the T_on_ of blend C15 was higher, but only by 23 °C. However, the temperatures corresponding to different weight losses of the polymer blends C5, C10 and C15 were also significantly increased compared to pure PH3B. The increase was by more than 35 °C compared to P3HB (236.2 °C). The increase in degradation temperature resulted from the presence of a more thermally stable component. A similar effect was observed in the cases of PHBV and thermoplastic polyurethane [30] and polyamide and P3HB [55]. The best improvement in thermal and mechanical properties has been observed for blends C5 and C10. The results regarding the thermal stability of the blends are consistent with the mechanical properties described earlier.

Under oxidation conditions (air atmosphere), the blends did not show any improvement in thermal stability compared to native P3HB (Table 7). All samples showed one-step degradation, while their Ton temperature values were similar, equaling 255 °C. The Ton temperature was about 4 °C higher in the case of C10 than during degradation in the nitrogen atmosphere. In turn, the temperatures of 5 wt. %, 10 wt. % and 50% were higher during degradation in nitrogen as an inert gas, and the values were about 7 to 11 °C higher in the case of nanocomposites. Similar observations have been made by other authors, e.g., Tidjani et al. for polypropylene–graft–maleic anhydride-nanocomposite [56], or Perepelkin et al. for aromatic fiber [57].

**Figure 17:**
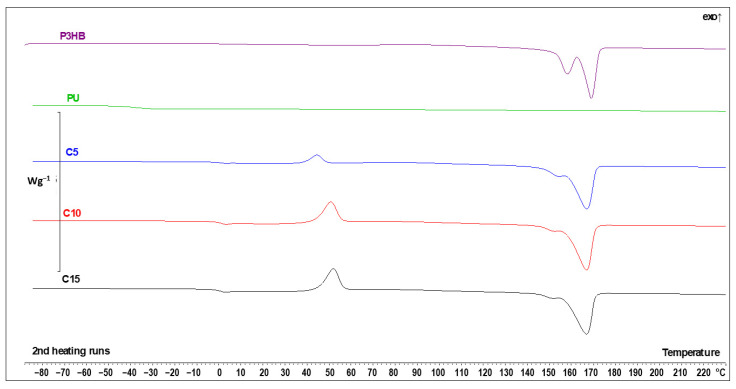
DSC thermal curves of P3HB, PU and the polymer blend with 5, 10 and 15 wt. % PU (C5, C10 and C15, respectively) upon heating at 10 °C·min^−1^ after prior cooling at the same rate.

The DSC data of virgin P3HB, PU and their blends are presented in Figure 17 and Table 8. The qualitative thermal analysis of blends identified the glass transition (Tg), cold crystallization and melting temperatures. PU showed a glass transition region and very low melting region, indicating PU is an amorphous material. Similarly, the P3HB thermogram showed glass transition and melting regions characteristic of semi-crystalline materials. The analysis of the glass transition region upon heating allowed us to determine the change of heat capacity (ΔCp) and the value of Tg. The analysis of melting area indicated the heat of fusion (ΔHf) and the beginning of melting Tm1(onset), Tm2(onset) and Tm3(onset). A clear trend was observed for all tested blend samples. It is seen that all the glass transition temperatures of the blends were similar and set in the range of 0.4 °C, so the differences can be considered as negligible. This confirms that the increase in the amount of PU added showed a very limited influence on the Tg values of the samples. The Tg of blends was decreased compared to the Tg of P3HB. This indicates the plasticizing effect of PU. A similar dependence was observed by Wang et al. [58] when P3HB was plasticized with dioctyl (*o*-)phthalate, dioctyl sebacate, and acetyl tributyl citrate.

The increase in the PU content is, however, important in the case of crystallinity considerations. For the sample with 5 wt. % of PU, the sample had a greater tendency to form a crystalline state during cooling. This is evidenced by both the crystallization enthalpy ΔHc and the change in the specific heat ΔCp in the glass transition process (the higher the ΔCp, the greater the fraction of amorphous phase, thus the smaller the crystalline fraction). Interestingly, there was no relevant difference in the cold crystallization process between C10 and C15 samples, but still, a lower PU content facilitated crystallization upon cooling, making the material more crystalline.

The PU sample showed a very-low-intensity melting process at around 130 °C, which is not visible in the C5-15 samples. Due to the low content of PU in those specimens, this can be considered as expected. The melting of P3HB is reflected in the mixtures of P3HB and PU, with lower melting enthalpies ΔHf with the decrease in P3HB content and slight shifts in the melting temperatures Tm_2_ and Tm_3_ to lower values due to PU addition, which has lower meting point and acts like some sort of contaminant. This is a typical behavior of P3HB, as also observed by Garcia-Garcia et al. via a decrease in the melting temperature for blends of P3HB and polycaprolactone [24].

Overall, there is a clear tendency towards a drop in crystallinity, and a tendency to crystallize during cooling with the increase in the PU content, what is partially compensated by cold crystallization. The glass transition temperature is influenced by PU addition, but with the limited possibility of the control of the Tg with changes in the PU concentration. Also, the melting of the native P3HB occurs at higher temperatures than in the mixtures due to the contamination-like action of the PU in the samples.

## 4. Conclusions

- New polymer blends based on poly(3-hydroxybutyrate) were prepared using linear polyurethane (PU) in amounts of 5, 10 and 15 wt. %. The polyurethane was synthesized by reacting 4,4′-diphenylmethane diisocyanate with polypropylene glycols with a molecular weight of 400 g. The preparation of bio-based blends was possible via direct mixing in a co-rotating twin screw extruder;
- The FTIR analysis confirmed the interaction between the components of the blends and their compatibility due to the formation of intermolecular hydrogen bonds between the P3HB and PU chains. The intensity of the interactions depended on the amount of PU in the polymer blend. The strongest interactions took place in the sample with 15 wt. % PU;
- It was observed that the most significant increase in surface roughness parameters occurred when P3HB was modified with 5 wt. % PU with respect to native P3HB, i.e., the strongest effect on the surface structure. An increase in the share of PU in the polymer blend resulted in an increase in surface roughness parameters, but this was not a linear increase. The surface of the blend had a more regular and dense roughness structure at higher levels of modification by PU;
- SEM measurements of the fracture surface of the blends subjected to destruction have allowed us to explain the mechanism of action of the polyurethane modifier applied on the mechanical properties of the blends. The introduction of the polyurethane modifier caused a disruption in the continuity of the polyester matrix structure. It caused the formation of rough domains separated by wavy areas resulting from the elasticizing effect of polyurethane, and this explains the decrease in hardness and increase in impact strength and elongation at break, especially for blends containing 5 and 10% PU by mass;
- The new P3HB-based blends exhibited a higher degradation temperature compared to the native P3HB. The polymer blend with 10 wt. % PU4 showed the largest difference in degradation temperature, which was 30 °C. The thermal stability of the rest of the blends was a bit worse, but still better than that of native P3HB;
- The thermal analysis of the heat parameters showed a decrease in the glass transition, melting and crystallization temperatures with increasing amounts of polyurethane in the blends. The decrease in the melting point of the blends is desired because the melting point of the native P3HB is not much higher than its decomposition temperature. The highest difference between the melting point and degradation temperature was measured for the blend with 10 wt. % polyurethane. The lower melting temperature of the resulting blends compared to native P3HB helped to improve the processing conditions and prevented the degradation of the material;
- The conducted tests prove the positive influence of polyurethane in the P3HB matrix on the thermal and mechanical properties of the prepared blends.

## Figures and Tables

**Table 1:** Compositions of extruded blends.

P3HB Content [wt. %]	PU Content, [wt. %]	Sample Determination
95	5	C5
90	10	C10
85	15	C15

**Table 2:** Extruder heating zone temperatures in the extrusion of polymer blends.

Sample	T_8_ [°C]	T_7_ [°C]	T_6_ [°C]	T_5_ [°C]	T_4_ [°C]	T_3_ [°C]	T_2_ [°C]	T_1_ [°C]
C5	160	160	160	160	160	160	155	140
C10	160	160	160	160	160	160	158	155
C15	156	156	156	156	156	160	153	149

**Table 3:** Results of molar masses and degrees of dispersion of the polyurethane obtained.

Mn [g/mol]	Mw [g/mol]	Mp [g/mol]	DI [–]
7612	17,588	28,132	2.31

**Table 4:** Roughness parameters of P3HB and its blends with 5, 10 and 15 wt. % PU, designated as C5, C10 and C15, respectively.

Sample	R_p_ [µm]	R_v_ [µm]	R_z_ [µm]	R_c_ [µm]	R_a_ [µm]	R_q_ [µm]	R_sm_ [µm]
P3HB	11.0	10.1	21.2	15.9	4.6	5.4	1400.9
C5	20.6	35.7	56.3	25.0	9.1	11.7	348.9
C10	18.0	20.3	38.3	15.2	4.2	5.9	312.5
C15	19.2	30	49.4	21.6	6.4	8.4	221.8

**Table 5:** Effect of polyurethane (PU) additive on wetting angles and surface characteristics of the P3HB–PU blends.

Sample	Average Wetting Angle (°)	Surface Characteristics
P3HB	72.14	Smooth, homogeneous structure
C5 (5% PU)	54.13	High roughness, pronounced irregularities
C10 (10% PU)	58.17	Moderate roughness, more balanced texture
C15 (15% PU)	62.23	Greater uniformity, more stable surface

**Table 6:** EDS analysis of P3HB microspheres and their blend with PU 5, 10 and 15 wt. % of PU, designated as C5, C10 and C15, respectively.

Sample	Element	Atomic %	Atomic % Error	Weight %	Weight % Error
P3HB	N	5.0	0.7	5.1	0.8
	C	51.0	0.3	44.1	0.3
	O	44.0	0.5	50.8	0.5
C5	N	5.1	0.8	5.1	0.8
	C	51.4	0.3	44.6	0.3
	O	43.5	0.5	50.3	0.5
C10	N	5.3	0.7	5.5	0.7
	C	51.5	0.3	44.5	0.3
	O	43.2	0.5	49.9	0.5
C15	N	5.6	0.8	5.7	0.8
	C	52.0	0.3	45.2	0.3
	O	42.4	0.5	49.1	0.6

**Table 7:** The interpretation of TG and DTG curves of pure P3HB and its blends with PU following a heating rate of 5 °C min^−1^ in a nitrogen and air atmosphere.

Sample	T_on_ [°C]	T_5% [_°C]	T_10%_ [°C]	T_50%_ [°C]	T_max_ [°C]	Residue at 400 °C [%]
nitrogen						
P3HB	221.1	236.2	245.6	281.2	291.7	1.41
C5	250.2	271.9	277.1	290.0	293.4	1.33
C10	251.2	275.2	281.5	295.2	293.7	1.52
C15	244.0	273.3	280.5	283.8	292.2	1.58
air						
P3HB	255.1	264.6	269.1	281.3	283.4	0.84
C5	255.6	264.1	268.3	279.3	281.4	2.24
C10	255.4	265.6	269.1	279.6	281.9	4.36
C15	255.8	264.3	268.3	279.1	280.9	6.01

**Table 8:** DSC results of P3HB, PU and their blends C5, C10 and C15 containing 5, 10 and 15 wt. % PU, respectively, upon heating the samples at the heating rate of 10 °C·min^−1^ after prior cooling at the same rate.

Sample	T_g1_, (°C)	ΔC_p_ (J·g^−1^·°C^−1^)	T_g2_, (°C)	ΔC_p_ (J·g^−1^·°C^−1^)	T_cc_, (°C)	ΔH_cc_ (J·g^−1^)	T_m1_, (°C)	ΔH_f1_ (J·g^−1^)	T_m2_, (°C)	T_m3_, (°C)	ΔH_f_ (J·g^−1^)	T_c_, (°C)	ΔH_c_, (J·g^−1^)
P3HB	-----	-----	5.5	0.148	89.9	−4.76	-----	-----	157.5	167.8	97.4	85.7	−78.6
PU	−38.7	0.517	-----	-----	-----	-----	131.6	0.38	-----	-----	-----	44.3	−0.38
C5	-----	-----	−0.5	0.205	44.5	−30.3	-----	-----	152.9	165.3	86.0	66.7	−55.5
C10	-----	-----	−0.6	0.414	50.9	−55.9	-----	-----	150.9	165.4	81.0	58.4	−24.8
C15	-----	-----	−0.9	0.424	52.1	−56.3	-----	-----	150.4	165.3	76.3	63.7	−19.9

## Data Availability

The original contributions presented in this study are included in the article. Further inquiries can be directed to the corresponding authors.
